# Polydatin Attenuates Hypoxic Pulmonary Hypertension and Reverses Remodeling through Protein Kinase C Mechanisms

**DOI:** 10.3390/ijms13067776

**Published:** 2012-06-21

**Authors:** Qing Miao, Xiao-Peng Shi, Ming-Xiang Ye, Jin Zhang, Shan Miao, Si-Wang Wang, Bo Li, Xiu-Xiu Jiang, Song Zhang, Nan Hu, Juan Li, Jian Zhang

**Affiliations:** 1 Institute of Materia Medica, Fourth Military Medical University, Xi’an 710032, China; E-Mails: miaoqing@fmmu.edu.cn (Q.M.); miaoshan@fmmu.edu.cn (S.M.); zhangsong801101@163.com (S.Z.); 2 Department of Pharmacy, Xijing Hospital, Fourth Military Medical University, Xi’an 710032, China; E-Mail: shixiaop@fmmu.edu.cn; 3 Department of Pulmonary Medicine, Xijing Hospital, Fourth Military Medical University, Xi’an 710032, China; E-Mails: mingxiangye@yahoo.cn (M.-X.Y.); shasha8502@yahoo.com (B.L.); jiangxuanlizf@163.com (X.-X.J.); 4 Department of Plastic Surgery, Xijing Hospital, Fourth Military Medical University, Xi’an 710032, China; E-Mail: jinzisay@fmmu.edu.cn; 5 Institute of Stomatology, General Hospital of People’s Liberation Army, Beijing 100853, China; E-Mail: beyondstephen@vip.sina.com; 6 Department of Physiology, Fourth Military Medical University, Xi’an 710032, China; E-Mail: lijuan2162008@163.com

**Keywords:** hypoxic pulmonary hypertension, remodeling, polydatin, protein kinase C

## Abstract

Hypoxic pulmonary hypertension is a life-threatening emergency if untreated. Consistent pulmonary hypertension also leads to arteries and ventricular remodeling. The clinical therapeutic strategy for pulmonary hypertension and the corresponding remodeling mainly interacts with NO, angiotensin II (Ang II) and elevated endothelin (ET) targets. In the present study, we evaluated the effects of polydatin on hypoxia-induced pulmonary hypertension. It was observed that polydatin attenuated hypoxic pulmonary hypertension, reversed remodeling, and regulated NO, Ang II, ET contents in the serum and lung samples. However, forced activation of PKC signaling by its selective activator thymeleatoxin (THX) could abate the effects of polydatain. These results suggest that polydatin might be a promising candidate for hypoxic pulmonary treatment through interaction with PKC mechanisms.

## 1. Introduction

Hypoxic pulmonary vasoconstriction as an adaptive process to severe oxygen shortage facilitates pulmonary capillary blood flow to alveolar ventilation and gas exchange, while prolonged excessive hypoxia and accompanying pulmonary capillary hypertension increase the incidence of fatal pulmonary edema. Excessive pulmonary vasoconstriction and vascular smooth muscle proliferation in chronic hypoxia lead to further increase in vascular resistance and to pulmonary hypertension. The most severe complication of consistent pulmonary hypertension is non-cardiac pulmonary edema. These patients manifest dyspnea, white or pink frothy sputum, moist rales on pulmonary auscultation, and flocculent shadows on chest X-rays. Hypoxic pulmonary hypertension also leads to psychological ailments in affected individuals. Therefore early detection and treatment of hypoxic pulmonary hypertension are of vitally essential. Supplement of oxygen is the most important treatment for hypoxic pulmonary hypertension. With increased understanding of the hypoxia-induced pulmonary hypertension mechanisms, elevated endothelin (ET) and angiotensin II (Ang II) in combination with reduced bioavailability of the endogenous vasodilator nitric oxide (NO) have been documented [1–3]. Various drugs hitting these targets (bosentan, losartan, silaenafil) have been clinically applied in the therapeutic strategy. There is also strong clinical evidence showing that glucocorticosteroid, calcium channel blockers, and other anti-inflammatory or vascular dilated agents are effective [4–7].

**Figure 1:**
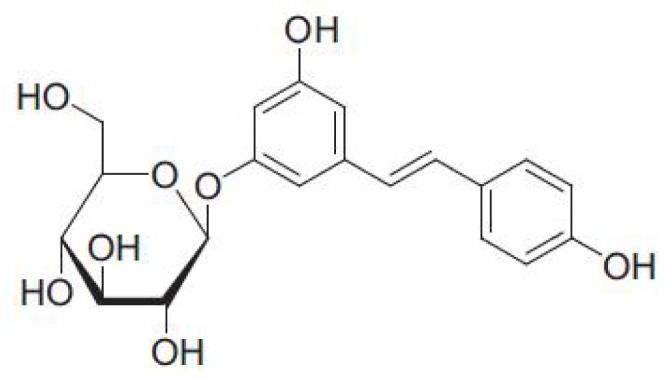
Chemical structure of polydatin (3,4′,5-trihydroxystilbene-3-β-mono-d-glucoside).

Polydatin (Figure 1), also termed 3,4′, 5-trihydroxystilbene-3-β-mono-d-glucoside, is a major active component derived from the plant *Polygonum cuspidatum*. An expanding body of studies has suggested that polydatin participates in many physiological processes, including inhibiting platelet aggregation, improving microcirculation, suppressing lipid peroxide, reducing neutrophil-endothelial cells adhesion, and anti-cancer activities [8–11]. Our recent study also revealed that polydatin preconditioning attenuates myocardial infarction during ischemia and reperfusion and limits the production of oxidants [12]. Likewise, other studies also showed that polydatin protects brain, intestine and remote organs against ischemia and reperfusion injury [13]. It is noted that aberrant protein kinase C (PKC) mechanisms during ischemia and reperfusion induce myocardial infarction and necrosis while polydatin communicates with these targets and restores myocardial function [12,14–17]. It is of special interest that changes in the proliferative potential of pulmonary artery smooth muscle cells (PASMCs) isolated from the hypertensive vessel wall as a response to hypoxia have been shown to be associated with significant changes in PKC activity [18,19]. However, whether polydatin attenuates pulmonary hypertension and whether polydatin interacts with PKC targets under the conditions of chronic hypoxia remains to be investigated. On the basis of our previous studies demonstrating a role for PKC targets as important contributors to the cardioprotective effect of polydatin during ischemia and reperfusion, we sought to investigate potential roles and the signaling pathway of polydatin in the animal model of hypoxia-induced pulmonary hypertension.

## 2. Results and Discussion

### 2.1. Hemodynamics during Hypopiesia and Hypoxia

We successfully established a rat hypoxic pulmonary hypertension model by both hypobaric and hypoxic methods. We observed that the rats developed pulmonary hypertension after three weeks of indicated treatment. Compared with the normoxic control group, chronic hypoxia induced a significant increase in mean pulmonary arterial pressure (mPAP) (* *p* < 0.05 *vs.* control). Pretreatment with the NO-donor drugs silaenafil markedly reduced the mPAP from (32.93 ± 3.08) mmHg to (26.08 ± 3.93) mmHg during hypopiesia and hypoxia (** *p* < 0.05 *vs.* hypoxia). Administration of polydatin dose-dependently reduced mPAP to (30.34 ± 2.19) mmHg, (27.71 ± 2.61) mmHg, and (25.21 ± 2.40) mmHg, respectively. The statistical difference was significantly in the 10 mg/kg polydatin and 20 mg/kg polydatin groups (Table 1).

As for the mean carotid arterial pressure (mCAP), there were no remarkable changes, suggesting that polydatin might selectively act on pulmonary vessels under the condition of hypopiesia and hypoxia.

### 2.2. Polydatin Attenuates Pulmonary Artery Remodeling and Right Ventricular Hypertrophy

Exposure to chronic hypoxia induced pulmonary artery remodeling and right ventricular hypertrophy as reflected by increased MT%, MA% and RV/(LV + S), RV/BW in rats, respectively (* *p* < 0.05 *vs.* control). It is shown in Table 2 that silaenafil not only reversed pulmonary artery remodeling but also attenuated right ventricular hypertrophy compared with the hypoxic animals (** *p* < 0.05 *vs.* hypoxia). Intraperitoneal administration of different doses of polydatin had similar actions as well, and its effects on pulmonary artery remodeling and right ventricular hypertrophy were dose-dependent (** *p* < 0.05 *vs.* hypoxia). These changes were more significant in the 10 mg/kg polydatin and 20 mg/kg polydatin groups, suggesting that polydatin at a relatively high dose might be an effective therapeutic agents for pulmonary hypertension.

### 2.3. Effects of Polydatin on Pulmonary Artery Morphology

**Figure 2:**
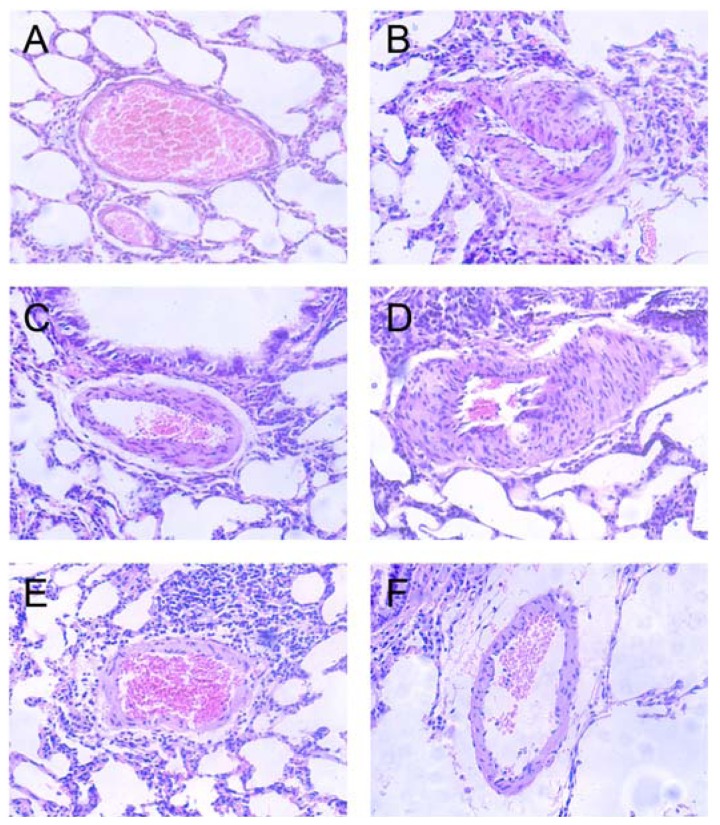
Effects of polydatin on pulmonary artery morphology during hypopiesia and hypoxia (HE staining, at 200× magnification). (**A**) normoxic group; (**B**) hypobaric and hypoxic group; (**C**) silaenafil group; (**D**) 5 mg/kg polydatin group; (**E**) 10 mg/kg polydatin group; (**F**) 20 mg/kg polydatin group.

Detected by light microscope, hypoxia promoted PASMCs proliferation and migration and led to endangium thickness (Figure 2A,B). Administration of silaenafil restrained these morphological changes after hypoxia (Figure 2C). The proliferation and migration of PASMCs as well as the thickness of the pulmonary artery wall were reduced significantly in hypoxic rats treated with 10 mg/kg polydatin and 20 mg/kg polydatin compared to the 5 mg/kg polydatin group (Figure 2D–F).

### 2.4. Polydatin Reverses Pulmonary Artery Remodeling

**Figure 3:**
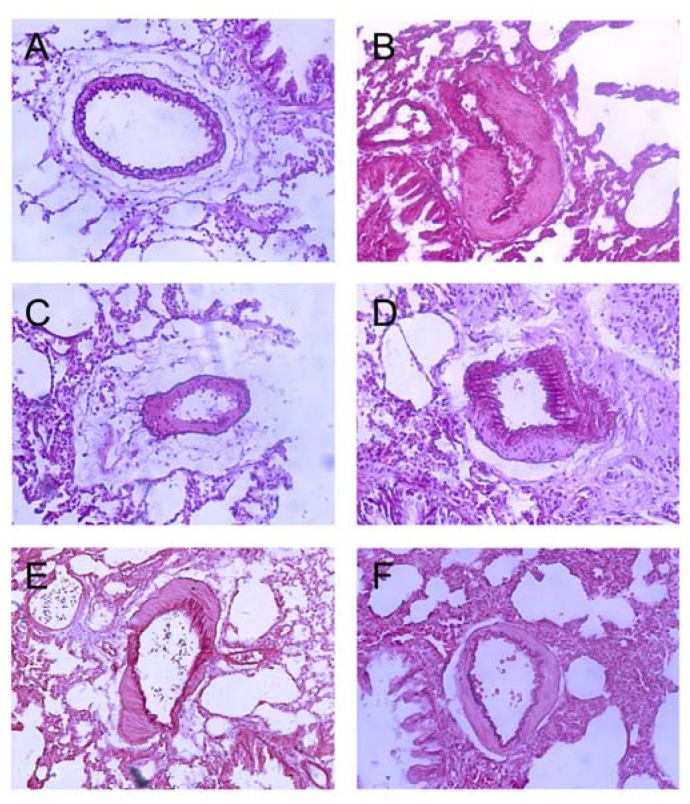
Van Gieson counterstaining showing elastic fibers during chronic hypoxia. (**A**) normoxic group; (**B**) hypobaric and hypoxic group; (**C**) silaenafil group; (**D**) 5 mg/kg polydatin group; (**E**) 10 mg/kg polydatin group; (**F**) 20 mg/kg polydatin group. (200× magnification).

The hyperplasia of elastic fibers as an early event of remodeling causes increased pulmonary vascular resistance [20]. Therefore, inhibiting the proliferation of elastic fibers is an important strategy for the prevention and treatment of pulmonary hypertension. To further evaluate the effects of polydatin on pulmonary artery remodeling, lung samples were subjected to van Gieson counterstaining to visualize the elastic fibers. It is noted chronic hypoxia resulted in the proliferation of elastic fibers, which could be attenuated by the NO-donor drug silaenafil (Figure 3A–C). Polydatin attenuated the proliferation of elastic fibers and reversed remodeling in hypoxic rats (Figure 3D–F), and this effect was especially significant in the high dose polydatin group (20 mg/kg).

### 2.5. Effects of Polydatin on NO, Ang II and ET

**Figure 4:**
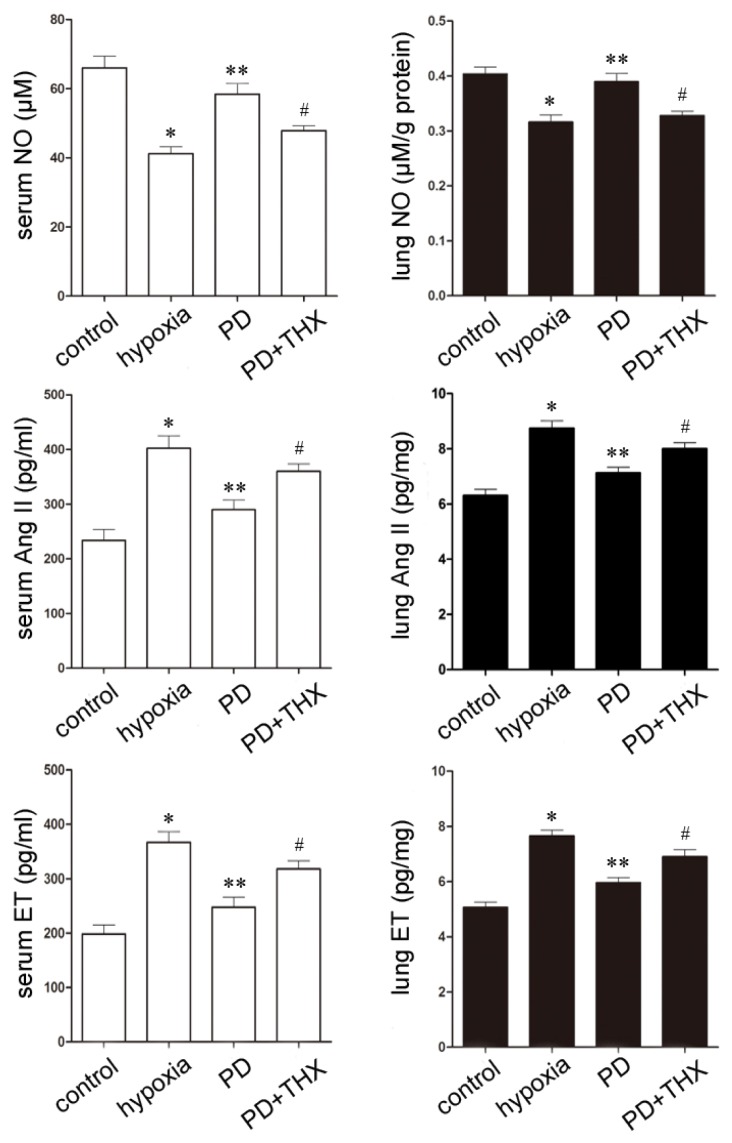
Effects of high dose polydatin on NO, Ang II and ET in the serum and lung samples. *n* = 8. Control: normoxic group; Hypoxia: hypobaric and hypoxic group; PD: 20 mg/kg polydatin group; PD + THX: 20 mg/kg polydatin and 0.2 mg/kg THX group. * *p* < 0.05 *vs.* control; ** *p* < 0.05 *vs.* hypoxia.

Several cytokines have been suggested to contribute to hypoxic pulmonary hypertension. The reduction of endogenous vasodilators (such as NO) and excess of vasoconstrictors (such as Ang II and ET) play critical roles in pulmonary vascular resistance and remodeling [21,22]. Since the preventive and therapeutic effects of polydatin on pulmonary hypertension are dose-dependent, as we have observed earlier, we herein investigate the effects of high dose polydatin (20 mg/kg) on these vasomotor factors during chronic hypoxia. Hypoxia stress upsets the balance of between NO, Ang II and ET, which triggers pulmonary hypertension and vascular remodeling characterized by hyperplasia of smooth muscle cells [23,24]. Consistent with previous studies, the contents of NO in both serum and lung tissue significantly decreased, while the level of Ang II and ET significantly increased during hypoxia [25]. We further found that 20 mg/kg polydatin increased the concentration of NO and decreased the concentration of Ang II and ET in both blood and pulmonary samples during hypoxia. The results indicate that polydatin may stimulate synthesis of NO in both blood and pulmonary tissue, and inhibit the production of Ang II and ET during hypoxia to play a role in the prevention of the development of hypoxic pulmonary hypertension (Figure 4).

### 2.6. Effect of Polydatin Is PKC-Dependent

There is abundant evidence to support that PKC signaling plays a crucial role in cardiovascular events. Upon activation, PKC causes pulmonary vasoconstriction and hypertension [26–28]. *In vitro* experiments showed that increased pulmonary vascular resistance and production of several cytokines are dependent on PKC, and inactivation of PKC signaling causes dilatation in isolated pulmonary arteries [19,29]. Meanwhile, we have reported in our previous study, that polydatin interacts with PKC signaling during myocardial ischemia and reperfusion [12]. It is of special interest to investigate whether polydatin attenuates pulmonary hypertension through inactivation of PKC signaling.

To test this idea, forced PKC signaling was activated by its selective activator thymeleatoxin (THX) [30]. THX treatment suppressed the effects of polydatin on serum NO, Ang II and ET concentrations also in the lung samples (Figure 4).

**Figure 5:**
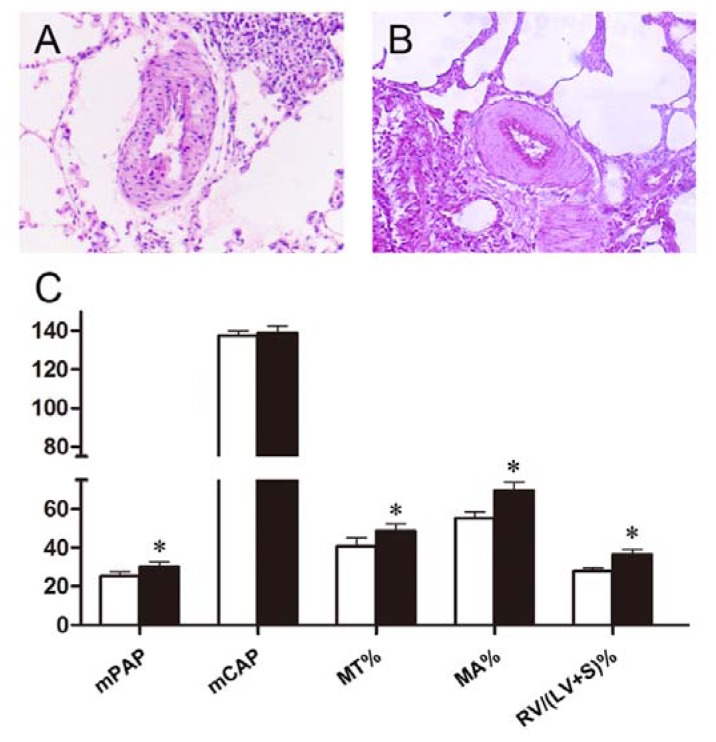
Effects of THX preconditioning on pulmonary hypertension and vascular remodeling in polydatin-treated rats. (**A**) Representative image of HE staining; (**B**) representative image of van Gieson counterstaining; (**C**) White column: 20 mg/kg polydatin group; Black column: THX preconditioning group. *n* = 8. * *p* < 0.05 *vs.* polydatin group.

We also found that THX preconditioning abolished potential therapeutic effect of polydatin on pulmonary hypertension and vascular remodeling. As we described above, polydatin inhibited PASMCs proliferation, migration and reversed pulmonary arteries remodeling in hypoxic rats (Figures 2 and 3). However, pretreatment with 0.2 mg/kg THX defected these beneficial effects of polydatin under hypoxic condition. On histological examination, forced PKC signaling by THX promoted hypoxic pulmonary hypertension and restrained the therapeutic effect of polydatin (Figure 5A). van Gieson counterstaining showed that THX preconditioning resulted in increased elastic fibers proliferation and pulmonary artery remodeling (Figure 5B).

Hemodynamic measurements also demonstrated similar findings (Figure 5C). We found that pretreatment with 0.2 mg/kg THX induced pulmonary hypertension and increased mPAP to (30.25 ± 0.20) mmHg in 20 mg/kg polydatin-treated rats. Changes in mCAP were not significant. However, effects of polydatin on pulmonary artery remodeling and right ventricular hypertrophy were suppressed by THX preconditioning. MT%, MA% and RV/(LV + S)% were increased to (48.73 ± 3.60)%, (69.85 ± 4.20)% and (36.52 ± 2.40)% as a response to THX, respectively.

Collectively, these results indicated that forced activation of PKC signaling by its selective activator THX could abolish therapeutic effects of polydatin on pulmonary hypertension and vascular remodeling under hypoxic condition. THX preconditioning also decreased vasodilator while increasing vasoconstrictor contents in polydatin-treated rats. These findings suggest that the PKC signaling pathway might play a crucial role in the development of pulmonary hypertension and vascular remodeling and might be a therapeutic target of polydatin.

## 3. Experimental Section

### 3.1. Reagents and Animals

Polydatin (PD), also termed 3,4′,5-trihydroxystilbene-3-β-mono-d-glucoside, was purchased from WeiJia Technology Company (Xi’an, China). Thymeleatoxin (THX, a PKC activator) was purchased from Sigma Chemical (St. Louis, MO, USA).

Adult male Sprague Dawley rats (250–300 g) obtained from the animal center of the Fourth Military Medical University were used for all experiments. This study conformed to the Guidelines for the Care and Use of Laboratory Animals published by the U.S. National Institutes of Health (NIH publication No. 85–23, revised 1985). All rats were exposed to a 12 h light-dark cycle and provided rat chow and water *ad libitum*.

### 3.2. Animal Groups and Models

Fourty eight male Sprague-Dawley rats were randomly divided into six groups: (1) normoxic group, normal control group; (2) low atmosphere and hypoxic group, the rats were intraperitoneally injected with 0.5 mL saline every other day followed by hypobaric and hypoxic conditions for 3 weeks; (3) positive control group, rats were intragastrically administrated 1.7 mg/kg silaenafil every other day 10 min prior to low atmosphere and hypoxia for 3 weeks; (4) low dose polydatin group, rats were intraperitoneally injected with 5 mg/kg polydatin every other day 10 min prior to low atmosphere and hypoxia for 3 weeks; (5) medium dose polydatin group, rats were intraperitoneally injected with 10 mg/kg polydatin every other day 10 min prior to low atmosphere and hypoxia for 3 weeks; (6) high dose polydatin group, rats were intraperitoneally injected with 20 mg/kg polydatin every other day 10 min prior to low atmosphere and hypoxia for 3 weeks. Hypobaric and hypoxic conditions were performed for 8 h every day by exposing rats to an automatic regulated low atmospheric pressure (50 kPa) and hypoxic (10% oxygen) chamber (Fourth Military Medical University). The normoxic group of rats was kept in room air.

### 3.3. Measurement of Hemodynamics and Right Ventricular Hypertrophy

After indicated treatments, rats were anesthetized with 30 g/L pentobarbital sodium (1.5 mL/kg, i.p.). A micro-catheter was inserted into the right ventricle and pulmonary artery through the right external jugular vein, and the mean pulmonary arterial pressure (mPAP), mean carotid arterial pressure (mCAP) and right ventricular pressure (RVP) were measured as previously described [31]. The right ventricle (RV), left ventricle (LV) and septum (S) were isolated, RV and LV + S were weighed. The RV/(LV + S) and RV/BW (body weight) ratios were calculated and used as indexes for right ventricular hypertrophy (RVH).

### 3.4. Pulmonary Artery Histology

Pulmonary artery morphology was performed as described previously [32,33]. Briefly, after fixation in 10% formalin (pH 7.4) for 1 week, the lung tissue was embedded using paraffin embedding and sliced serially. The tissues were stained with HE (hematoxylin and eosin) and elastic fibers staining (Har’t elastic fibers staining improving method, van Gieson counterstain). Morphologic changes of the peripheral pulmonary artery were detected with a light microscope, and the microscopic images were analyzed using a computerized morphometric system. The external diameter (ED), medial-wall thickness (MT), medial cross-sectional area (MA), vessel lumen cross-sectional area (VA) and total arterial cross-sectional area (TAA) of the peripheral pulmonary artery were measured. The ratio of vascular medial wall thickness to external diameter (MT%) and the ratio of vascular medial cross-sectional area to total arterial cross-sectional area (MA%) were calculated to assess the degree of pulmonary artery remodeling.

### 3.5. Forced Activation of PKC

To further investigate the underlying mechanisms of polydatin-mediated pulmonary capillaries diastolic and remodeling reversing effects, the PKC signaling was activated by its activator THX. THX (0.2 mg/kg) was intravenously administered 10 min before polydatin treatment followed by hypobaric and hypoxic conditions.

### 3.6. Serum NO, ET and Ang II Assays

Vasoactive substances in the serum such as NO, ET and Ang II were measured as described [21,22]. Three milliliters of arterial blood was collected, blood serum was separated, and NO level was determined by NO assay kit (Jiancheng Company, Nanjing) (calculated NO_2_^−^/NO_3_^−^ by the standard curve law, NO = NO_2_^−^/NO_3_^−^). The blood was centrifuged at 3000 rpm (10 min at 4 °C), the plasma was separated, and ET and Ang II levels were measured by ET and Ang II radio immunoassay kits according to the manufacturers’ instructions (Center of Technology Exploitation in PLA General Hospital, Beijing).

### 3.7. Lung Tissue NO, ET and Ang II Assays

At the end of the experiment, the same positions of the inferior lobe of the right lung were taken, and tissues were homogenized, then the homogenate were centrifuged, and the suspensions were collected for the examination of NO, ET and Ang II levels as described [21,22].

### 3.8. Statistical Analysis

Data are expressed as means ± SD. Differences between groups were analyzed by one-way analysis of variance (ANOVA) followed by *LSD* post hoc test using SPSS statistical software (SPSS, Inc., Chicago, IL, USA). Significance was considered at * *p* < 0.05.

## 4. Conclusions

Polydatin prevents hypoxic pulmonary hypertension and reverses remodeling under hypobaric and hypoxic conditions. Polydatin also regulates the synthesis and release of NO, Ang II and ET, which contribute to pulmonary resistance and remodeling. The PKC activator THX could attenuate these effects of polydatin on hypoxic pulmonary hypertension rats, suggesting that the mechanism of action for polydatin lies in its interaction with PKC signaling. Collectively, polydatin might be a promising therapeutic strategy for hypoxic pulmonary hypertension.

## Figures and Tables

**Table 1:** Effects of polydatin on mean pulmonary arterial pressure (mPAP) and mean carotid arterial pressure (mCAP) in rats exposed to chronic hypoxia. *n* = 8.

	mPAP (mmHg)	mCAP (mmHg)
control	18.74 ± 1.74	138.05 ± 3.55
hypoxia	32.93 ± 3.08 *	140.15 ± 5.81
silaenafil	26.08 ± 3.93 **	138.20 ± 2.77
5 mg/kg PD	30.34 ± 2.19	139.48 ± 4.27
10 mg/kg PD	27.71 ± 2.61 **	138.72 ± 3.22
20 mg/kg PD	25.21 ± 2.40 **	137.67 ± 4.53

* *p* < 0.05 *vs.* control;

** *p* < 0.05 *vs.* hypoxia.

**Table 2:** Effects of polydatin on pulmonary artery remodeling and right ventricular hypertrophy in chronic hypoxic rats. *n* = 8. MT: medial wall thickness; MA: media cross-sectional area; RV: right ventricle; LV: left ventricle; S: septum; BW: body weight.

	MT%	MA%	RV/(LV + S)%	RV/BW (mg/g)
control	31.63 ± 2.66	43.54 ± 3.17	22.20 ± 1.21	0.56 ± 0.08
hypoxia	50.72 ± 4.50 *	72.99 ± 4.47 *	37.67 ± 2.57 *	0.92 ± 0.14 *
silaenafil	39.28 ± 5.26 **	49.84 ± 6.34 **	25.57 ± 2.57 **	0.63 ± 0.13 **
5 mg/kg PD	46.27 ± 3.88	68.85 ± 3.26	34.23 ± 1.92 **	0.80 ± 0.17
10 mg/kg PD	45.01 ± 4.25 **	58.85 ± 4.74 **	30.63 ± 1.44 **	0.75 ± 0.13 **
20 mg/kg PD	40.75 ± 4.38 **	55.27 ± 3.41 **	27.87 ± 1.48 **	0.73 ± 0.12 **

* *p* < 0.05 *vs.* control;

** *p* < 0.05 *vs.* hypoxia.
